# Beyond Swelling: Clinical Insights into the Diagnosis and Management of Hereditary Angioedema

**DOI:** 10.3390/ijms27177680

**Published:** 2026-08-27

**Authors:** Margarita Paulauskiene, Laura Tamasauskiene, Brigita Gradauskiene

**Affiliations:** 1 Department of Immunology and Allergology, Lithuanian University of Health Sciences, Eiveniu Str. 2, LT-50009 Kaunas, Lithuania; margarita.paulauskiene@lsmu.lt (M.P.); brigita.gradauskiene@lsmu.lt (B.G.); 2 Laboratory of Immunology, Department of Immunology and Allergology, Lithuanian University of Health Sciences, Eiveniu Str. 2, LT-50009 Kaunas, Lithuania

**Keywords:** hereditary angioedema, angioedema, swelling, C1 esterase inhibitor deficiency, bradykinin

## Abstract

Hereditary angioedema (HAE) is a rare genetic disorder characterized by recurrent episodes of severe, non-pitting edema, most commonly caused by a deficiency or dysfunction of C1 esterase inhibitor (C1-INH), resulting in excessive bradykinin production. HAE subtypes differ according to C1-INH levels, functional activity, underlying genetic variants, and increasingly recognized molecular mechanisms. Awareness of HAE outside specialized medical fields remains limited, and recurrent angioedema may arise through different pathophysiological mechanisms, which together contribute to frequent misdiagnosis, particularly in patients without a positive family history. Consequently, many patients experience significant diagnostic delays and inappropriate interventions. Rapid advances in endotype-based classification, molecular diagnostics, and targeted therapies have substantially changed the understanding and management of HAE, highlighting the need for an updated clinical overview. This review provides a comprehensive overview of HAE classification, clinical presentation, genetic background, diagnosis, and current management. Particular attention is given to advances in diagnostic biomarkers and genetic testing, newly approved targeted therapies, and oral on-demand treatment. Together, these advances are moving HAE management toward earlier diagnosis, more individualized treatment, and more patient-centered care.

## 1. Introduction

Hereditary angioedema (HAE) is a group of rare genetic disorders characterized by recurrent episodes of swelling involving subcutaneous and submucosal tissues. HAE is most commonly inherited as an autosomal dominant disorder caused by a deficiency or dysfunction of C1 esterase inhibitor (C1-INH), a key regulator of the complement and plasma kallikrein–kinin systems. C1-INH belongs to the serine protease inhibitor (serpin) superfamily and is synthesized primarily in hepatocytes. Its main function is to regulate activation of the complement cascade and prevent excessive bradykinin production, which is responsible for increased vascular permeability and angioedema formation [1]. Although the exact global prevalence of HAE remains uncertain, recent evidence suggests that HAE affects approximately 1–2 individuals per 100,000 people worldwide. A recent systematic review and meta-analysis including 24 studies and 11,245 HAE cases estimated a pooled global prevalence of 1.22 cases per 100,000 population (95% CI, 0.91–1.53) [2]. Reported prevalence was lower in Asia and Africa than in Europe and North America, highlighting notable geographical variation in HAE epidemiology [2]. Regional differences in reported prevalence may partly reflect underdiagnosis, limited disease awareness, and disparities in access to diagnostic testing rather than true differences in disease occurrence [3]. Diagnosis may be challenging because different forms of angioedema can present with similar clinical manifestations despite having distinct pathophysiological mechanisms. Unlike mast cell-mediated angioedema associated with urticaria, HAE is not accompanied by wheals or pruritic skin rash and typically presents as non-pitting edema. Delayed or inaccurate diagnosis may lead to recurrent debilitating attacks, impaired quality of life, and potentially life-threatening complications. Since rapid diagnostic tests to reliably differentiate bradykinin-mediated from mast cell-mediated angioedema are not currently available, careful evaluation of clinical presentation, attack characteristics, and family history remains essential for accurate diagnosis and timely treatment [4].

This narrative review provides an updated overview of HAE, with particular emphasis on its evolving endotype-based classification, molecular and genetic background, clinical presentation, diagnostic and differential diagnostic approaches, and contemporary management. Particular attention is given to recent therapeutic advances, including newly approved targeted therapies, oral on-demand treatment, and emerging RNA-based and gene-editing strategies.

## 2. Literature Search and Selection Criteria

Scope of the Review. The review focused on recent advances in HAE classification, molecular pathophysiology, genetics, clinical presentation, diagnosis, differential diagnosis, and therapeutic management, with particular attention to newly approved and investigational therapies and current international recommendations.

Eligibility and Source Selection. English-language peer-reviewed publications published between 2000 and August 2026 were included if they addressed the predefined topics of the review, including HAE classification, molecular pathophysiology, genetics, clinical presentation, diagnosis, differential diagnosis, or treatment. Eligible publications included original clinical and experimental studies, clinical trials, systematic reviews and meta-analyses, international guidelines, consensus documents, and relevant narrative reviews.

Search Strategy. Studies were searched for in the PubMed database. Medical Subject Headings (MeSH) and related text terms were used, including “Hereditary Angioedema,” “C1 Inhibitor,” “Bradykinin,” “Kallikreins,” “Genetic Testing,” and “Angioedema.” Additional targeted searches were performed for specific therapeutic agents, molecular targets, and HAE-associated genetic variants. As this was a narrative review, publications were selected according to their relevance to the predefined topics. A total of 38 articles were included in the review.

## 3. HAE Pathophysiology and Classification

The pathogenesis of hereditary angioedema (HAE) varies according to the specific disease subtype. Previous international WAO/EAACI guidelines for the management of HAE recognized three major forms of the disease: type I HAE, characterized by low antigenic and functional C1 esterase inhibitor (C1-INH) levels; type II HAE, caused by dysfunctional C1-INH; and HAE with normal C1-INH levels (HAE-nC1-INH) [5].

**Figure 1:**
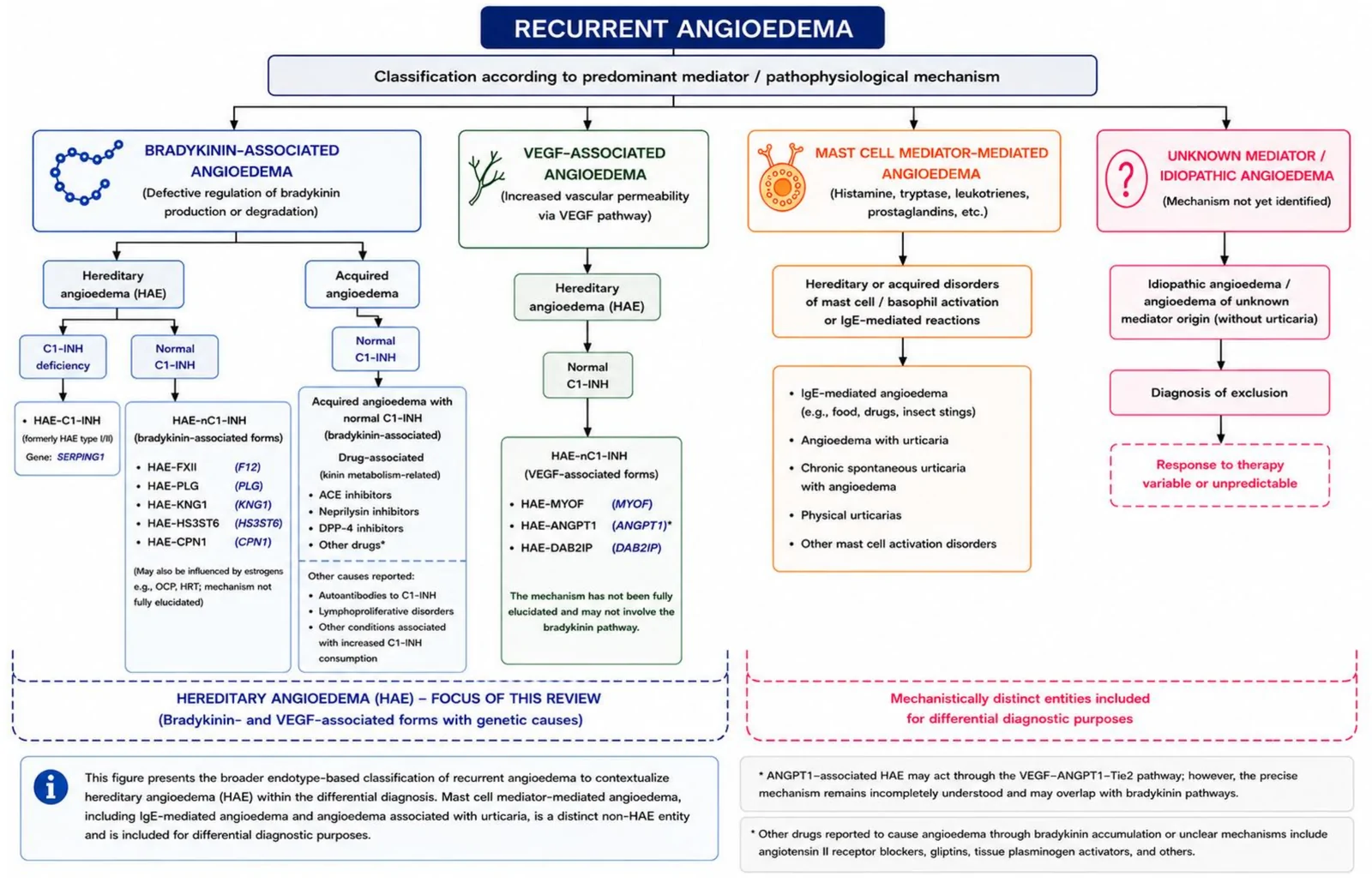
Proposed classification of recurrent angioedema based on underlying endotypes. The figure illustrates the broader mechanistic classification of recurrent angioedema and includes hereditary as well as acquired, drug-related and mast cell mediator-induced forms; the latter should not be interpreted as HAE subtypes. Figure generated using AI-assisted tools and modified by the authors. Adapted from: Vázquez et al. World Allergy Organization Journal (2026) 19:101335 [6]. Abbreviations: HAE: hereditary angioedema; C1-INH: C1 inhibitor; VEGF: vascular endothelial growth factor; HAE-C1-INH: HAE with C1-INH deficiency; HAE-nC1-INH: HAE with normal C1-INH; HAE-ANGPT1: HAE with angiopoietin-1 (*ANGPT1*) mutation; HAE-CPN1: HAE with carboxypeptidase N (*CPN1*) mutation; HAE-DAB2IP: HAE with DAB2-interacting protein (*DAB2IP*) mutation; HAE-FXII: HAE with factor 12 (*F12*) mutation; HAE-HS3ST6: HAE with heparan sulfate-3-O-sulfotransferase 6 (*HS3ST6*) mutation; HAE-KNG1: HAE with kininogen 1 (*KNG1*) mutation; HAE-MYOF: HAE with myoferlin (*MYOF*) mutation; HAE-PLG: HAE with plasminogen (*PLG*) mutation.

Since HAE types I and II share similar pathophysiological mechanisms, clinical manifestations, and management strategies, recent guidelines propose a simplified classification using the unified term hereditary angioedema with C1 inhibitor deficiency (HAE-C1-INH) [5,6]. The traditional distinction between these two forms primarily reflects the nature of the C1-INH defect: type I is characterized by reduced antigenic and functional C1-INH levels, whereas type II typically involves normal or elevated antigenic levels of dysfunctional C1-INH. Despite this biochemical distinction, both forms are associated with pathogenic variants in *SERPING1* and ultimately result in insufficient functional C1-INH activity, leading to dysregulation of the plasma contact system, excessive kallikrein activity, and increased bradykinin generation [7]. In contrast, the term HAE type III, originally used to describe patients with hereditary angioedema and normal C1-INH levels and function, has been replaced by hereditary angioedema with normal C1 inhibitor (HAE-nC1-INH). Ongoing advances in the understanding of the genetic and molecular mechanisms underlying hereditary angioedema (HAE) have highlighted the limitations of classifications based predominantly on clinical phenotype and C1-INH status. Accordingly, updated WAO recommendations proposed an endotype-based classification that incorporates the pathophysiological mechanisms underlying recurrent angioedema. This framework distinguishes bradykinin-mediated angioedema, vascular endothelial growth factor (VEGF)-associated angioedema, mast cell-mediated angioedema (including angioedema associated with urticaria), and idiopathic angioedema or angioedema of unknown mediator origin [6]. Drug-associated angioedema with normal C1-INH may result from impaired bradykinin degradation or altered kinin metabolism. Angiotensin-converting enzyme (ACE) inhibitors are the best-established cause, while angiotensin II receptor blockers, neprilysin inhibitors, dipeptidyl peptidase-4 (DPP-4) inhibitors, and tissue plasminogen activators have also been associated with angioedema. The strength of evidence and underlying mechanisms vary among these drug classes [5,6]. By distinguishing biologically different forms of angioedema that may present with similar clinical manifestations, this approach may support more accurate diagnosis, appropriate treatment selection, and genetic counseling. Importantly, the proposed classification reflects current scientific evidence and represents an evolving framework that may be refined as new molecular and mechanistic data emerge. A schematic representation of this classification is presented in Figure 1.

C1-INH regulates several interconnected plasma proteolytic systems, including the classical complement pathway, the contact system, intrinsic coagulation, and the plasminogen–plasmin system [8]. Persistent bradykinin generation due to inadequate inhibition of factor XIIa and plasma kallikrein is the principal mechanism underlying angioedema formation in HAE [8]. In addition, plasma kallikrein-mediated cleavage of factor XII may promote activation of the complement cascade, including generation of the complement fragments C4a and C5a, which have been suggested to contribute to increased endothelial permeability. Cytokines also play an important role in HAE pathogenesis. During acute attacks, increased levels of proinflammatory cytokines, including interleukin (IL)-1, IL-4, IL-6, IL-8, and IL-13, further enhance endothelial permeability. Although current evidence supports a central role of the bradykinin B2 receptor in angioedema development, upregulation of the bradykinin B1 receptor during stress, trauma, or infection may influence susceptibility to attacks. Furthermore, prolonged B1 receptor signaling may contribute to sustained edema during HAE episodes [9].

At the endothelial level, bradykinin signaling through B2R activates phospholipase C (PLC), protein kinase C (PKC), intracellular Ca^2+^ mobilization, and RhoA-dependent pathways [10]. Experimental studies have shown that bradykinin and kallikrein induce VE-cadherin cleavage and the release of soluble VE-cadherin fragments, accompanied by disruption of endothelial cell–cell junctions and increased vascular permeability [11]. Kinin receptor activation also stimulates endothelial nitric oxide production, further modulating vascular permeability [10,12]. B1R and B2R differ in their regulation: B2R undergoes rapid desensitization, whereas B1R is induced by inflammatory stimuli and exhibits slower and incomplete desensitization [10,12]. These mechanistic insights also provide the molecular rationale for current and emerging targeted therapies directed against key components of the kallikrein–kinin pathway, including plasma kallikrein, activated factor XII, prekallikrein synthesis, and bradykinin B2 receptor signaling.

Endothelial barrier integrity is additionally influenced by VEGF and ANGPT1–Tie2 signaling. ANGPT1–Tie2 signaling promotes endothelial barrier stability, whereas VEGF signaling favors junctional disruption and vascular leakage [10,12]. These pathways are particularly relevant to HAE subtypes associated with variants in *ANGPT1* and *MYOF*, suggesting that altered endothelial signaling, rather than excessive bradykinin generation alone, may contribute to disease pathogenesis [12].

Hereditary angioedema with normal C1 inhibitor levels (HAE-nC1-INH), previously referred to as type III HAE, is currently recognized as a heterogeneous group of very rare disorders. At present, several subtypes of HAE-nC1-INH have been identified based on underlying genetic mutations [5,7].

Acquired angioedema due to C1 inhibitor deficiency (AAE-C1-INH) is typically characterized by recurrent angioedema episodes occurring in patients without a positive family history, most commonly after the age of 40 years [13]. Unlike hereditary forms, acquired angioedema occurs sporadically as a result of excessive C1-INH consumption and is typically diagnosed in middle-aged or older individuals [5].

Accurate classification distinguishing HAE with C1-INH deficiency, HAE with normal C1-INH, and acquired forms of angioedema may be further supported by genetic testing [7].

Pathways important in C1-inhibitor deficiency in HAE may also play a role in autoimmunity. A Swedish population-based cohort study demonstrated that patients with HAE were at increased risk of autoimmune disease compared with individuals in the general population [14].

Patients who do not exhibit any currently identified genetic mutations or known mediator abnormalities, despite having a similar clinical presentation, are classified as having idiopathic angioedema. It is anticipated that additional pathogenic mutations will be identified in this subgroup of patients in the future.

## 4. Genetic Background

Several genetically defined forms of HAE have been described; however, not all causative genetic variants have been identified. HAE-C1-INH is associated with pathogenic variants in *SERPING1*, the gene encoding C1-INH [6]. The *SERPING1* gene exhibits marked allelic heterogeneity, with 809 of 893 reported variants (90.5%) classified as pathogenic or likely pathogenic [15]. The mutational spectrum includes missense, nonsense, frameshift, and splice-site variants, as well as small insertions/deletions and larger genomic rearrangements [15]. De novo pathogenic variants have also been reported and may explain the absence of a family history in a subset of patients with HAE-C1-INH [15,16]. Pathogenic *SERPING1* variants can impair the synthesis, secretion, or function of C1-INH, resulting in insufficient regulation of the plasma contact system, excessive activation of plasma kallikrein and factor XII (FXII), cleavage of high-molecular-weight kininogen, and increased bradykinin generation [7].

Despite the extensive genetic heterogeneity of *SERPING1*, the relationship between genotype and clinical phenotype remains incompletely defined. Earlier studies suggested potential genotype–phenotype associations, including a milder clinical phenotype in patients carrying certain missense *SERPING1* variants [17]. Studies have reported substantial variability in disease severity and clinical course, even among members of the same family carrying the same pathogenic variant, suggesting that the *SERPING1* genotype alone may not fully predict disease expression [7]. Epigenetic, environmental, and other disease-modifying factors have therefore been proposed to contribute to the observed phenotypic variability [7].

More recent studies combining advanced genetic sequencing with functional characterization of C1-INH variants have sought to further elucidate the relationship between specific *SERPING1* alterations and their molecular and clinical consequences. Using whole-exome and whole-genome sequencing together with recombinant protein expression and functional analyses, Ren et al. demonstrated that individual *SERPING1* variants may affect C1-INH through distinct mechanisms, including impaired protein synthesis, folding, and inhibitory function. The authors also suggested that specific variant combinations may contribute to a more severe clinical phenotype [18]. These findings indicate that genotype–phenotype relationships in HAE-C1-INH are complex and highlight the value of integrating genetic data with functional characterization to better understand interindividual variability in disease expression.

Efforts to identify the underlying genetic causes of HAE-nC1-INH have led to the identification of several genetically defined subtypes. To date, disease-associated variants have been identified in genes encoding coagulation factor XII (*F12*), angiopoietin-1 (*ANGPT1*), plasminogen (*PLG*), kininogen-1 (*KNG1*), myoferlin (*MYOF*), and heparan sulfate-glucosamine 3-O-sulfotransferase 6 (*HS3ST6*). More recently, variants in the genes encoding carboxypeptidase N (*CPN1*) and DAB2-interacting protein (*DAB2IP*) have also been reported in association with HAE-nC1-INH [19].

Pathogenic variants in the coagulation factor XII gene (*F12*) were the first genetic alterations identified in patients with HAE-nC1-INH [19]. These variants have been reported most frequently in families from Brazil, France, Spain, Turkey, and Germany, with fewer cases identified in other populations. Notably, known HAE-associated pathogenic *F12* variants have not been identified in Japanese HAE-nC1-INH families studied to date, suggesting potential population-specific differences in the genetic background of HAE-nC1-INH [19]. Genotype-associated differences in clinical expression have also been observed. HAE-FXII shows a marked female predominance and is strongly influenced by estrogen exposure, with disease onset or worsening frequently associated with estrogen-containing contraceptives or pregnancy. In contrast, HAE-PLG is characterized by a particular tendency toward facial and tongue swelling, with tongue involvement representing an important clinical feature because of the potential risk of upper airway obstruction [19].

Genetic testing can complement biochemical evaluation of HAE. In HAE-C1-INH, diagnosis is primarily based on biochemical testing, and genetic testing is generally not required; however, identification of a pathogenic variant in *SERPING1* may confirm the molecular diagnosis in cases of diagnostic uncertainty [6]. In patients with a clinical phenotype suggestive of HAE but normal C1-INH antigenic levels and functional activity, molecular testing plays a key role in identifying genetically defined forms of HAE-nC1-INH. Nevertheless, a negative genetic test does not exclude HAE-nC1-INH, as additional disease-associated genes and pathogenic variants may remain unidentified [15,19].

## 5. Clinical Presentation

The clinical expression of HAE varies widely among patients and does not necessarily align with the levels of C1-inhibitor protein or its function in the blood [19,20]. Physical signs of HAE include overt, noninflammatory swelling of the skin and mucous membranes. Sites of involvement include the subcutaneous tissues, abdominal organs, and upper airway [21]. Angioedema attacks are typically unpredictable, focal, asymmetric, and potentially disfiguring. Although swelling may simultaneously involve multiple anatomical sites, attacks are generally localized rather than systemic [6].

Erythema marginatum (EM) is an important prodromal manifestation and typically presents as a transient, non-raised, erythematous, usually non-pruritic rash and may therefore be mistaken for urticaria, potentially contributing to diagnostic delay [5,22]. Unlike many prodromal symptoms, which are subjective and nonspecific, EM represents an objective and clinically recognizable sign that may indicate an impending angioedema attack. In a Japanese cohort of patients with HAE-C1-INH, 53.4% had experienced EM [22]. Among 31 patients with HAE who experienced EM, 71.0% reported neither itching nor tingling, and the forearm was the most frequently affected site (64.5%), followed by the abdomen (29.0%). The interval between recognition of EM and subsequent angioedema varied considerably, ranging from 0 to 48 h [22]. Erythema marginatum has been reported in approximately 42–58% of pediatric patients [23].

In pediatric patients, HAE-C1-INH generally presents with recurrent subcutaneous and/or submucosal edema similar to that observed in adults, although clinical manifestations are uncommon during infancy. The reported age of symptom onset varies considerably, from approximately 4.4 to 18 years, with a mean age at the first attack of around 10 years and no clear difference between boys and girls. Earlier disease onset has been associated with a more severe subsequent disease course, while attack frequency and severity may increase during puberty, likely reflecting hormonal influences [23]. Subcutaneous edema involving the extremities, face, trunk, or genital region is generally the most common and often the earliest manifestation in children and may persist for 1–5 days before resolving spontaneously. Abdominal involvement may manifest as recurrent severe colicky pain, nausea, vomiting, diarrhea, and, in more severe attacks, hypovolemia; because abdominal pain is common in childhood, these attacks may remain unrecognized as a manifestation of HAE. Upper airway involvement usually occurs later and is less common in children; however, because of the smaller airway diameter, progressive upper airway edema may lead to rapid obstruction and requires immediate recognition and treatment [1,23].

Clinical manifestations, diagnostic implications, and potential complications of hereditary angioedema according to the affected site of HAE are presented in Table 1.

The attacks may last 2 to 5 days, usually slowly increasing and then resolving even without therapy [1]. In a cohort of 123 patients with HAE, the mean interval from the onset of laryngeal symptoms to maximum edema development was 8.3 h [24]. The frequency and duration of HAE attacks vary considerably between patients. In the absence of effective treatment, attacks may occur approximately every 7–14 days on average [6]. Commonly reported triggers include trauma, infections, stress, and medical or dental procedures. For women with HAE and normal C1-INH, attacks may be triggered or exacerbated by high estrogen levels [4]. In the absence of an HAE diagnosis, the risk of death with laryngeal edema is estimated at 20–25% [6].

### Diagnosis and Differential Diagnosis

As angioedema may present as a medical emergency in some cases, identifying its underlying cause can be challenging, particularly during initial episodes. It is helpful to assess the symptom onset rate, age at first symptoms, skin changes during the attack, previously taken medications, as well as response to antihistamines and epinephrine if they were administered.

Several clinical screening tools have been developed to facilitate earlier recognition of hereditary angioedema. The ABC warning signs for HAE diagnosis include A—angioedema, B—bradykinin, C—C1 inhibitor deficiency, D—distress factor, E—epinephrine nonresponsiveness, F—family history, and G—gastrointestinal and/or glottic edema [25].

The diagnosis of HAE is established based on clinical presentation, family history, and laboratory evaluation of complement system proteins, including C1 inhibitor levels and functional activity [20,26]. According to the international WAO/EAACI guidelines, joint assessment of serum antigenic C4 and C1-INH levels and plasma functional C1-INH is advised. When all three tests are used together, the diagnostic accuracy for HAE-C1-INH is very high and significantly greater than that of any single test alone [5,26]. C3 levels are expected to be normal in HAE, and testing is not helpful [5]. In HAE-C1-INH, the form previously known as type I is characterized by decreased C1-INH antigenic levels (<50% of the normal mean) and reduced functional activity, whereas the form previously known as type II is characterized by normal or elevated C1-INH antigenic levels but reduced functional activity. Because C1-INH testing is performed infrequently in many laboratories, there is an increased risk of both false-positive and false-negative results, which can be reduced through repeat testing. C4, C1-INH antigenic level, and C1-INH functional activity are typically normal in HAE-nC1-INH and ACE inhibitor-induced angioedema. In AAE-C1-INH, C1-INH antigenic levels may be decreased or normal, whereas C1-INH functional activity and C4 are typically decreased. Detection of autoantibodies against C1 esterase inhibitor (C1-INH-Ab), including assessment of C1-INH autoantibody concentration (CAC), may provide valuable additional diagnostic information alongside complement panel evaluation in patients with acquired angioedema due to C1 inhibitor deficiency (AAE-C1-INH) [27]. Many patients with AAE-C1-INH have reduced C1q levels and autoantibodies that inactivate C1-INH [13].

Early identification of pediatric hereditary angioedema with C1 inhibitor deficiency is essential; however, several factors regarding complement system protein levels during the first year of life must be considered. Complement levels in umbilical cord blood of full-term neonates are physiologically lower than maternal levels due to immaturity of the complement system. In newborns, both antigenic and functional C1 inhibitor (C1-INH) levels correspond to approximately 60–70% of adult values. Premature infants exhibit even lower quantitative and functional C1-INH levels compared with full-term neonates. In most cases, C1-INH levels reach adult values between 6 months and 1 year of age. Therefore, complement testing performed before this period should be repeated after the age of one year [23].

It is very important to differentiate HAE from other forms of angioedema. The major hallmarks that distinguish histamine-mediated angioedema from HAE are urticaria/wheals, rapid symptom onset, and a medical history revealing potential allergen exposure. In contrast to histaminergic angioedema, antihistamines, corticosteroids, and epinephrine have limited efficacy in HAE; thus, treatment involves agents that inhibit bradykinin production or antagonize its receptor [21,28]. The most important laboratory diagnostic patterns of bradykinin-mediated angioedema are summarized in Table 2.

Angiotensin-converting enzyme inhibitors (ACE-Is) are a well-established cause of acquired bradykinin-mediated angioedema due to impaired bradykinin degradation. Other drugs that may be associated with bradykinin-mediated angioedema include dipeptidyl peptidase-4 (DPP-4) inhibitors (gliptins), neprilysin inhibitors, and, less consistently, angiotensin II receptor blockers (ARBs) and tissue plasminogen activators. The involvement of bradykinin in ARB-associated angioedema remains controversial [20,28].

ACE inhibitor-associated angioedema should always be considered in any patient taking an ACE inhibitor who experiences angioedema. Over the past several years, the incidence of ACE-I-induced angioedema has increased significantly, and ACE inhibitors are currently used by an estimated 40 million patients [28].

In patients with biochemical evidence of C1-INH deficiency or dysfunction, particularly in the absence of a positive family history and with disease onset later in life, AAE-C1-INH should be considered and excluded [5,20]. The principal distinguishing clinical and laboratory features are summarized in Table 3.

Several potential biomarkers have been proposed; however, none have yet been fully validated for routine clinical use [19]. Reliable biomarkers of kallikrein–kinin system activation may aid in distinguishing the underlying mechanisms of angioedema. Emerging assays for high-molecular-weight kininogen (cHMWK), activated plasma kallikrein, and FXIIa show promise as biochemical tools for identifying bradykinin-mediated angioedema. Inter-α-trypsin inhibitor heavy chain 4 (ITIH4) was also investigated as a novel biomarker for HAE diagnosis and monitoring [20].

## 6. Treatment

Advances in understanding the pathophysiology of HAE have been fundamental to progress in the field, particularly the recognition of the kallikrein–kinin system—namely, plasma kallikrein and activated factor XII—as a central therapeutic target. This insight has driven the development of novel therapies, several of which are now approved for the treatment of HAE [20,26].

Treatment is based on the following main methods: treatment of acute angioedema attacks (on-demand treatment), short-term (preprocedural) prophylaxis, and long-term prophylaxis [5,6]. Non-pharmacological management of hereditary angioedema focuses on patient education, avoidance of known triggers (e.g., trauma, stress, infections, and estrogen exposure), avoidance of precipitating medications such as ACE inhibitors, and ensuring preparedness for medical or dental procedures and emergency situations [5].

**Early on-demand treatment (ODT)** of hereditary angioedema attacks results in a better therapeutic response than delayed treatment; therefore, HAE attacks should be treated as early as possible. Early intervention is associated with faster symptom resolution and a shorter overall attack duration, regardless of attack severity [5,6].

Specific medications used for the treatment of acute attacks in patients with HAE are intravenous (IV) plasma-derived nano-filtered and purified human C1-INH concentrate (pdC1-INH), IV recombinant human C1-INH (RhC1-INH), subcutaneous (SC) icatibant acetate, and SC ecallantide [1,5,26]. Early ODT of HAE attacks with intravenous C1-INH, ecallantide or icatibant provides a better treatment response than late treatment. Icatibant, a 10-amino acid synthetic peptide, is a specific and selective competitive antagonist of the bradykinin B2 receptor and prevents binding of bradykinin to its receptor. It has a plasma half-life of 1–2 h. Most attacks resolve after a single injection of icatibant [5,29]. The available oral ODT is sebetralstat, a competitive, reversible inhibitor of plasma kallikrein, and the dose is 2 tablets of 300 mg [6].

When first-line therapies are unavailable, acute attacks should be managed with solvent/detergent-treated plasma (SDP). In settings where SDP is not accessible, fresh frozen plasma (FFP) may be used. Fresh frozen plasma is usually avoided due to allosensitization unless one of the previous drugs is not available. The use of antifibrinolytic agents (e.g., tranexamic acid) or androgens for on-demand treatment of HAE attacks is not recommended [5,6].

Plasma-derived C1 inhibitor (pdC1-INH) is produced from cryodepleted human plasma through sequential adsorption and precipitation, purification, pasteurization, and viral filtration processes. Two pdC1-INH concentrates are currently available for on-demand treatment (ODT) of hereditary angioedema attacks. Because pdC1-INH replaces the deficient or dysfunctional C1 inhibitor, it can be used both for the treatment of acute attacks and for short-term prophylaxis prior to medical, surgical, or dental procedures. The mean plasma half-life of pdC1-INH exceeds 30 h [7,26]. When pdC1-INH is administered, the recommended dose is 20 IU/kg, rounded to the nearest 500 IU because the product is supplied in 500-IU vials, thereby minimizing product waste [6].

Recombinant human C1-INH (RhC1-INH) is identical in its mode of action to that of pdC1-INH. RhC1-INH is produced through genetic engineering, and because it is secreted in the milk of transgenic rabbits, it is contraindicated in patients with a known allergy to rabbits [6]. RhC1-INH is indicated for on-demand treatment of all types of HAE attacks in adults and children (2 years or older) [5,23].

The kallikrein inhibitor ecallantide is licensed only in the United States and a few Latin American countries for the on-demand treatment of all types of HAE attacks in HAE-C1-INH patients aged 12 years and older. It blocks bradykinin generation by inhibiting kallikrein and preventing amplification via FXIIa. Ecallantide is a 60-amino-acid recombinant protein with a plasma half-life of approximately 2 h; its main safety concern is hypersensitivity, including anaphylaxis, reported in 3–4% of patients [6,30].

**Short-term prophylaxis (STP),** or preprocedure prophylaxis, was first described as the treatment administered before medical or surgical procedures to prevent angioedema episodes [5]. Angioedema associated with these procedures usually occurs within 48 h. Currently, it also includes preventive treatment before vital events (e.g., exams, weddings) and during especially stressful life periods (e.g., divorce), which can elicit angioedema episodes [21,26]. Intravenous plasma-derived C1 inhibitor (pdC1-INH) is recommended as first-line short-term prophylaxis. Accordingly, preprocedural prophylaxis with intravenous pdC1-INH concentrate is recommended for all medical, surgical, and dental procedures involving mechanical manipulation of the upper aerodigestive tract. According to current recommendations, pdC1-INH concentrate should be administered intravenously as close as possible to the procedure, preferably less than 6 h beforehand. Although the optimal dosage has not been fully established and approved indications may vary by country, most experts recommend either a fixed dose of 1000 units or a weight-based dose of 20 units/kg of pdC1-INH [5,31].

FFP may be used for short-term prophylaxis but is considered a second-line option due to lower safety compared with intravenous pdC1-INH concentrate and increased risks of blood-borne infection and allosensitization. Attenuated androgens (e.g., danazol) were previously used for preprocedural prophylaxis but are no longer preferred, as intravenous pdC1-INH concentrate is the agent of choice, and repeated short courses of androgens may cause cumulative adverse effects. Tranexamic acid has also been used historically but is not recommended by most guidelines. Icatibant acetate and ecallantide are not advised for use as STP due to their short half-life and the lack of evidence [5].

Preprocedural prophylaxis in pregnancy is recommended, preferably with C1-INH, for interventions that come with a risk of attacks such as chorionic villus sampling, amniocentesis, and induced surgical abortion [5]. Administering C1-INH concentrate as preprocedural prophylaxis is recommended before labor and delivery when symptoms have been recurring frequently during the third trimester and the patient’s history includes genital edema caused by mechanical trauma during forceps delivery or vacuum extraction. Vaginal delivery is preferred because surgery or general anesthesia may involve endotracheal intubation. For cesarean section, preprocedural prophylaxis with C1-INH and epidural anesthesia is recommended, with intubation avoided if possible [5].

### Long-Term Prophylaxis (LTP)

Recent advances in long-term prophylactic therapies have substantially reduced attack frequency, with many patients achieving a complete response. LTP is a continued or maintenance treatment to decrease the frequency, severity, and duration of angioedema attacks [1].

Since the publication of updated international guidelines, additional targeted therapies, including garadacimab and donidalorsen, have been incorporated into first-line long-term prophylactic treatment options for patients with HAE-C1-INH, alongside plasma-derived SC-C1-INH, lanadelumab, and berotralstat [5,6].

Lanadelumab is a fully human monoclonal antibody (IgG1/κ light chain) administered subcutaneously that inhibits active plasma kallikrein. Due to its efficacy and subcutaneous route of administration, it is one of the preferred agents for long-term prophylaxis to prevent HAE attacks. It is typically administered as 300 mg every 2 weeks; however, a dosing interval of 300 mg every 4 weeks may be considered if a patient is well controlled (e.g., attack-free) [32]. Long-term treatment with lanadelumab has been shown to reduce the mean HAE attack rate by 87.4% overall [9].

Berotralstat is a plasma kallikrein inhibitor that binds to plasma kallikrein and suppresses its proteolytic activity. Because of its effectiveness and oral formulation, it is also considered a preferred option for long-term prophylaxis of HAE attacks. It is typically administered as 150 mg orally with food, with dose reductions to 110 mg in some regions where it is licensed, based on whether there is hepatic impairment, use of P-glycoprotein or BCRP inhibitors (drug interactions) or whether patients experience gastrointestinal symptoms on the 150-mg dose [33].

**Figure 2:**
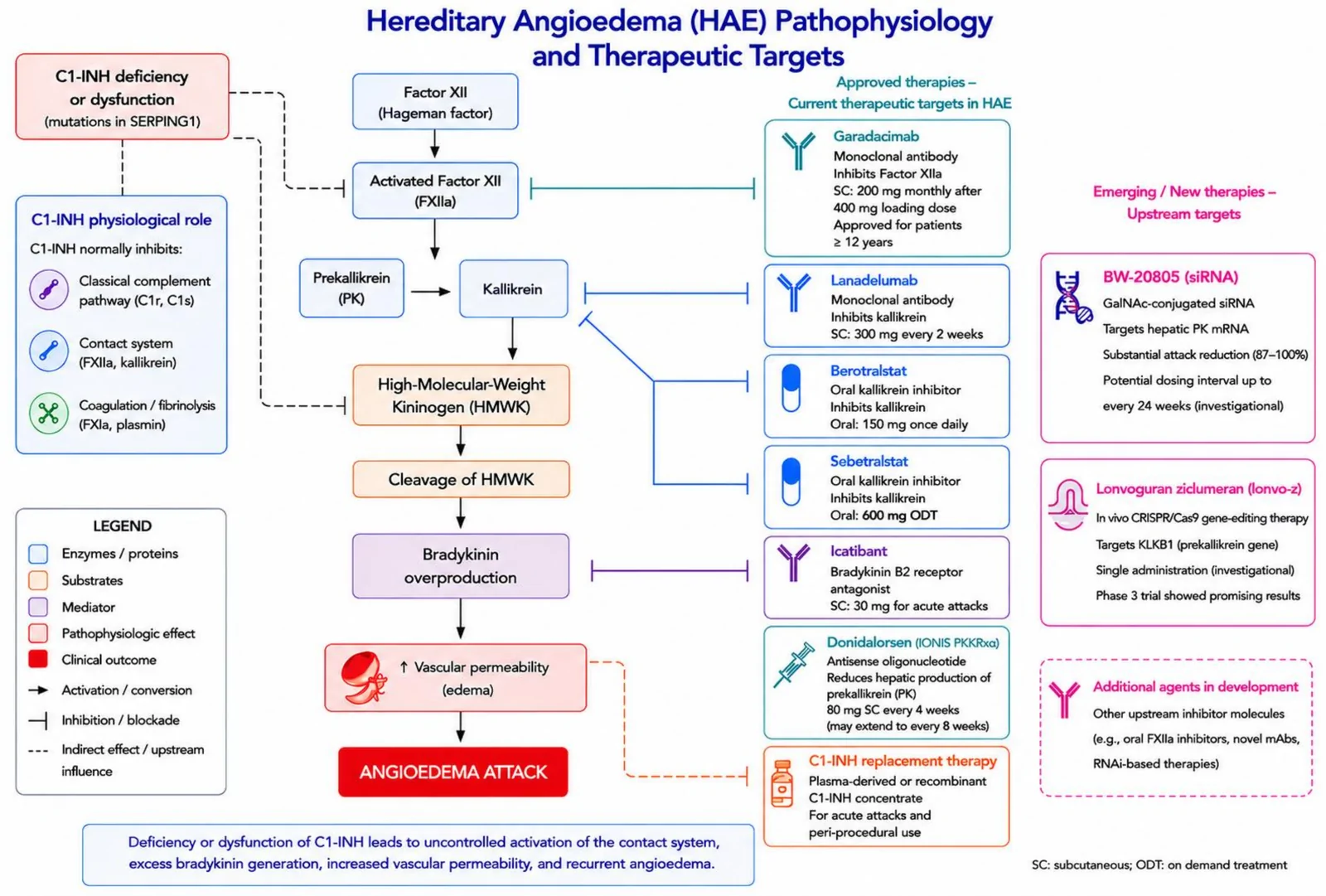
Hereditary angioedema pathophysiology and therapeutic targets. Figure generated using AI-assisted tools and modified by the authors. Regulatory status: garadacimab, sebetralstat, and donidalorsen are approved in the United States (FDA) and authorized in the European Union (EMA/EC), according to their respective indications.

Although garadacimab and donidalorsen represent relatively novel therapeutic agents, they have already been incorporated into the latest international recommendations as first-line options for LTP in patients with HAE [6,20,26]. Garadacimab is a fully human monoclonal antibody administered subcutaneously once monthly at a dose of 200 mg. Treatment is initiated with a loading dose of 400 mg administered as two 200 mg injections, resulting in a rapid onset of action. Garadacimab selectively inhibits activated factor XIIa at the top of the kallikrein–kinin pathway. It is generally well tolerated, with minimal injection-site reactions reported, and can be used in children aged 12 years and older [34]. Donidalorsen is an antisense oligonucleotide that suppresses hepatic production of prekallikrein, which is primarily synthesized in the liver. It is administered subcutaneously at a dose of 80 mg monthly, but may be extended out to every 8 weeks [35]. The pathophysiologic mechanisms and therapeutic targets of hereditary angioedema are presented in Figure 2.

Therapies that are now less commonly used include antifibrinolytics and attenuated androgens. Previously, attenuated androgens (e.g., danazol, stanozolol), synthetic derivatives of testosterone, were used for long-term prophylaxis of hereditary angioedema. They increase hepatic synthesis of C1-INH, thereby reducing the frequency and severity of angioedema attacks. However, their use is currently limited due to frequent adverse effects, including hyperlipidemia, obesity, androgenic effects in females, and mood disturbances. These issues are primarily caused by residual hormonal activity and tend to be more pronounced in women [5,26].

Antifibrinolytics, including tranexamic acid and aminocaproic acid, have limited effectiveness in the prevention and treatment of attacks. Tranexamic acid competitively inhibits the activation of plasminogen, with a reduction in the transformation of plasminogen into plasmin and a decrease in fibrinolysis. The dose ranges from 1000 to 3000 mg/d divided into 2–3 oral administrations per day [26]. Tranexamic acid has limited efficacy in HAE-C1-INH and is generally considered a second-line option for long-term prophylaxis when first-line therapies are unavailable, not feasible, or not tolerated. It may also be considered in selected situations, including childhood or pregnancy [6].

Emerging prophylactic strategies for HAE increasingly focus on suppression of the kallikrein–kinin pathway using siRNA-based therapies. BW-20805, a chemically modified GalNAc-conjugated siRNA targeting hepatic plasma prekallikrein expression, represents a promising long-acting therapeutic approach. Preliminary results from an ongoing Phase 2 study published in 2026 demonstrated a substantial reduction in HAE attack rates (87–100%), with the majority of treated patients remaining attack-free during the observation period [36]. BW-20805 was generally well tolerated, with mainly mild transient injection-site reactions reported. Sustained plasma prekallikrein suppression suggests the potential feasibility of extended dosing intervals of up to every 24 weeks [36].

Recent advances in HAE treatment have also introduced gene-editing approaches targeting the kallikrein–kinin pathway. Lonvoguran ziclumeran (lonvo-z), an investigational in vivo CRISPR-based gene-editing therapy targeting *KLKB1*, demonstrated significant efficacy in the Phase 3 HAELO trial. A single 50-mg intravenous dose reduced the monthly HAE attack rate by 87% compared with placebo, with no serious or grade ≥3 adverse events reported in the lonvo-z group during the 28-week trial period [37].

In HAE, complete disease control is generally considered the absence of clinically significant attacks. Long-term prophylaxis should be individualized and considered for all patients with HAE-C1-INH, taking into account disease activity, patient quality of life, healthcare-resource availability, and inadequate disease control despite appropriate on-demand therapy. Patients should be assessed for the need for long-term prophylaxis at each visit, considering disease activity, disease burden, disease control, and patient preferences. Optimal HAE management should focus not only on preventing attacks but also on restoring normal quality of life [6]. Non-pharmaceutical management strategies for patients with HAE are summarized in Table 4.

The expanding range of therapeutic options allows HAE management to be increasingly individualized according to treatment indication, attack burden, route and frequency of administration, patient preferences, and treatment availability. A comparative overview of current HAE therapies, including dosing regimens, mechanisms of action, major advantages and limitations, and their current place in therapy, is provided in Table 5.

## 7. Discussion and Limitations

Recent advances in the understanding of HAE have substantially changed both disease classification and therapeutic management. The transition toward an endotype-based classification reflects increasing recognition that recurrent angioedema encompasses mechanistically heterogeneous disorders and that not all genetically defined forms of HAE with normal C1-INH can yet be attributed exclusively to bradykinin-mediated pathways. At the same time, the therapeutic landscape has expanded rapidly toward increasingly upstream and mechanism-directed interventions, allowing treatment to be individualized according to disease burden, route and frequency of administration, patient preference, and treatment availability.

Several limitations of this review should be acknowledged. First, this is a narrative rather than a systematic review; therefore, study selection was based on relevance to the predefined topics, and formal systematic-review methodology, risk-of-bias assessment, and quantitative evidence synthesis were not performed. Second, the evidence base is heterogeneous across HAE subtypes. Most therapeutic evidence and current management recommendations relate to HAE-C1-INH, whereas data for genetically defined HAE-nC1-INH remain substantially more limited. Furthermore, the molecular mechanisms underlying several HAE-nC1-INH subtypes remain incompletely understood, particularly regarding the relative contributions of bradykinin-mediated and VEGF-associated pathways.

Finally, the HAE therapeutic landscape is changing rapidly. Several recently approved therapies have comparatively limited long-term and real-world experience, while emerging approaches, including RNA-based and gene-editing therapies, remain under clinical investigation. Regulatory approval, availability, and indications may also differ between regions. Consequently, some aspects of classification and treatment discussed in this review are likely to evolve as additional mechanistic, long-term safety, comparative-effectiveness, and real-world data become available.

## 8. Conclusions

HAE is a rare, complex, and unpredictable lifelong disease with a substantial impact on patients and their families. Earlier recognition of bradykinin-mediated angioedema remains essential to reduce diagnostic delay, avoid ineffective treatment, and prevent potentially fatal upper airway compromise. Diagnosis of HAE-C1-INH relies primarily on the combined assessment of C4 concentration and C1-INH antigenic level and functional activity, while genetic testing is particularly important in diagnostically uncertain cases and in identifying genetically defined forms of HAE-nC1-INH.

Advances in molecular understanding have expanded the traditional concept of HAE beyond C1-INH deficiency and support an evolving endotype-based classification. In parallel, therapeutic management has progressed from C1-INH replacement and bradykinin receptor blockade toward increasingly targeted approaches, including inhibition of plasma kallikrein and activated factor XII and suppression of prekallikrein synthesis. Importantly, the introduction of sebetralstat as the first oral on-demand therapy represents a major advance in acute HAE management, providing a convenient, needle-free treatment option and potentially facilitating earlier treatment of attacks. Current first-line long-term prophylactic options—including plasma-derived SC C1-INH, lanadelumab, berotralstat, garadacimab, and donidalorsen—provide greater opportunities to achieve sustained disease control and individualize treatment according to attack burden, patient preferences, route and frequency of administration, safety, access, and quality of life.

Emerging RNA-based and gene-editing strategies further illustrate the transition toward long-acting, mechanism-directed, and potentially disease-modifying treatment. However, these therapeutic advances also highlight persistent disparities in access to HAE care. The high cost and unequal availability of newer targeted therapies may substantially influence treatment selection and limit access to optimal care, particularly in resource-constrained settings. Therefore, future progress in HAE management should encompass not only the development of more effective and convenient therapies, but also efforts to improve affordability and equitable access to treatment. Together, advances in disease classification, molecular diagnosis, effective and convenient on-demand therapy, individualized prophylaxis, and improved treatment accessibility are moving HAE management toward more precise, patient-centered, and equitable care.

## Figures and Tables

**Table 1:** Clinical manifestations, diagnostic implications, and potential complications of hereditary angioedema according to the affected site [1,4,6].

Affected Site	Typical Clinical Manifestations	Diagnostic Implications	Potential Complications/Severity
Subcutaneous tissues	Non-pruritic, non-pitting swelling involving the face, hands, arms, legs, genitals, and buttocks	Facial or peripheral swelling may be mistaken for allergic or mast cell-mediated angioedema	Pain, discomfort, and temporary functional impairment; facial/oral swelling may coexist with or precede upper airway involvement
Gastrointestinal tract	Bowel-wall edema causing severe, often colicky abdominal pain, nausea, vomiting, and diarrhea; ascites may occur	May mimic an acute surgical abdomen, potentially leading to unnecessary diagnostic or surgical procedures	Pain, dehydration, ascites and hypovolemia in extensive attacks
Upper airway/oral cavity	Swelling of the tongue, pharynx, or larynx; dysphagia, voice changes, throat tightness, and stridor may occur	Requires prompt recognition as bradykinin-mediated angioedema because conventional treatment for mast cell-mediated angioedema may be ineffective	Potentially life-threatening upper airway obstruction and asphyxiation
Genitourinary tract	Genital swelling; bladder or urethral involvement may cause urinary symptoms	May be mistaken for infectious, inflammatory, or allergic conditions	Pain, functional impairment, and, rarely, urinary obstruction

**Table 2:** Laboratory Diagnostic Patterns of Bradykinin-Mediated Angioedema [6,7,8,23].

Bradykinin-Mediated Angioedema Subtype	C1-INH Antigenic Level	C1-INH Functional Activity	C4	C1q	Anti-C1-INH	Associated Genes	Role of Genetic Testing
HAE-C1-INH ^a^	Decreased	Decreased	Decreased (80–95% of cases)	Normal	Absent	*SERPING1*	Not routinely required; may confirm the diagnosis in uncertain cases
HAE-C1-INH ^b^	Normal or elevated	Decreased	Decreased (80–95% of cases)	Normal	Absent	*SERPING1*	Not routinely required; may confirm the diagnosis in uncertain cases
HAE-nC1-INH	Normal	Normal	Normal	Normal	Absent	*F12*, *PLG*, *ANGPT1*, *KNG1*, *MYOF*, *HS3ST6*, *CPN1*, *DAB2IP*	Important for identifying genetically defined subtypes
AAE-C1-INH	Decreased or normal	Decreased	Decreased	Decreased (approximately 75% of cases)	Present (approximately 50% of cases)	-	Not routinely indicated
ACE inhibitor-induced angioedema	Normal	Normal	Normal	Normal	Absent	-	Not routinely indicated

^a^ Previously referred to as HAE type I. ^b^ Previously referred to as HAE type II. Abbreviations: AAE-C1-INH, acquired angioedema with C1 inhibitor deficiency; C1-INH, C1 inhibitor; HAE-C1-INH, hereditary angioedema with C1 inhibitor deficiency; HAE-nC1-INH, hereditary angioedema with normal C1 inhibitor.

**Table 3:** Differential diagnosis of angioedema [4,21,28].

Clinical Feature	Hereditary Bradykinin-Mediated Angioedema (HAE)	Acquired C1-INH Deficiency (AAE-C1-INH)	ACE Inhibitor-Induced Angioedema	Mast Cell-Mediated Angioedema
Triggers/associated factors	Trauma, procedures, infections, stress, estrogen exposure; may occur spontaneously	Often associated with lymphoproliferative disorders, monoclonal gammopathy, or autoimmune disease	ACE inhibitor exposure	Foods, medications, infections, physical triggers; may occur spontaneously
Family history	Often present, but may be absent	Usually absent	Absent	Usually absent
Typical age/onset	Usually childhood or adolescence	Typically after 40 years of age	May occur at any time during ACE inhibitor treatment	Any age
Onset of swelling	Gradual, usually over hours	Gradual, usually over hours	Usually gradual, over hours	Usually more rapid
Duration	2–5 days if untreated	Usually prolonged	Usually prolonged compared with mast cell-mediated angioedema	Usually shorter than bradykinin-mediated angioedema
Urticaria (wheals)	Absent	Absent	Absent	Often present, but may be absent
GI involvement	Common	May occur	Uncommon	Uncommon
Upper airway involvement	May occur; potentially life-threatening	May occur; potentially life-threatening	Face, lips, tongue, and upper airway commonly involved	May occur, particularly in anaphylaxis
Response to antihistamines, glucocorticoids, epinephrine	No or minimal response	No or minimal response	No or minimal response	Usually responsive

**Table 4:** Comprehensive Supportive Management for Patients with Hereditary Angioedema [5,6,38].

Stage of Care	Management Measure	Key Clinical Message
At diagnosis	Patient and caregiver education	Explain the disease, inheritance, common triggers, and principles of attack recognition and management; provide education on appropriate on-demand treatment and self-administration.
	Family screening and genetic counseling	Recommend cascade family testing using C1-INH testing or genetic testing, as appropriate according to age and HAE subtype; provide genetic counseling when appropriate.
Prevention and risk reduction	Trigger identification and avoidance	Identify individual triggers. Common triggers include trauma, medical/dental procedures, stress, infections, and estrogen exposure; attacks may also occur spontaneously.
	Medication review	Avoid ACE inhibitors and estrogen-containing medications when appropriate and review medications that may increase angioedema risk.
	Vaccination and infection prevention	Maintain routine infection-prevention measures, including recommended influenza and COVID-19 vaccination; consider hepatitis A/B vaccination in patients regularly receiving plasma-derived products or FFP.
Emergency preparedness	Individual emergency plan and access to on-demand treatment	Ensure that patients have an individualized emergency plan and access to effective on-demand treatment; educate patients/caregivers in self-administration.
	Medical identification	Provide appropriate documentation or medical identification to facilitate rapid recognition of HAE in emergency settings.
Procedural planning	Pre-procedural risk assessment	Consider short-term prophylaxis before procedures associated with increased attack risk, including dental procedures, upper-airway manipulation, and surgery requiring intubation.
Long-term follow-up	Regular assessment of disease control	Reassess disease activity, attack burden, treatment effectiveness, and health-related quality of life regularly and adjust management through shared decision-making.
	Psychological and quality-of-life support	Assess the psychosocial and quality-of-life burden of HAE and provide appropriate support when needed.
	Healthcare coordination	Maintain clear communication and documentation across specialist, emergency, dental, and surgical care.

**Table 5:** Comparative overview of current treatment options for hereditary angioedema (HAE) [5,6,26,31].

Treatment Category	Therapy	Route/Dose/Frequency	Mechanism/Target	Major Advantages	Key Limitations/Considerations	Current Place in Therapy
On-demand treatment	Plasma-derived C1-INH	IV; 20 IU/kg	C1-INH replacement	Established efficacy; all ages	IV access; plasma-derived	First-line ODT
	Recombinant C1-INH	IV; 50 IU/kg, max 4200 IU	C1-INH replacement	No human plasma exposure	IV administration	First-line ODT
	Icatibant	SC; 30 mg adults; pediatric 0.4 mg/kg, max 30 mg	Bradykinin B2 receptor antagonist	Rapid SC self-administration	Injection-site reactions; repeat dose may be required	First-line ODT
	Sebetralstat	Oral; 600 mg (2 × 300 mg)	Plasma kallikrein inhibitor	Oral ODT; supports early treatment	New therapy; limited long-term real-world evidence	First-line ODT
	Ecallantide	SC; 30 mg	Plasma kallikrein inhibitor	Direct kallikrein inhibition	Risk of hypersensitivity; supervised use	Second-line ODT
	Solvent/detergent-treated plasma	IV; 200 mL	Provides functional C1-INH	Alternative where specific ODT unavailable	Plasma-related risks	Third-line ODT
	Fresh frozen plasma (FFP)	IV; 400 mL	Provides functional C1-INH	May be available when specific therapy unavailable	Transfusion-related risks	Third-line ODT
Short-term prophylaxis	Plasma-derived C1-INH	IV; 1000 U or 20 IU/kg before procedure	C1-INH replacement	Preferred preprocedural prophylaxis	Requires IV access; timing close to procedure	First-line STP
	Recombinant C1-INH	IV; 50 IU/kg, max 4200 IU	C1-INH replacement	No human plasma exposure	Less established for STP in some settings	Alternative STP
	Fresh frozen plasma (FFP)	IV; preprocedural use when needed	Provides functional C1-INH	Accessible in some settings	Lower safety; infection/allosensitization risk	Second-line STP
	Solvent/detergent-treated plasma	IV; preprocedural use when needed	Provides functional C1-INH	Preferred plasma option over FFP	Availability varies	Alternative STP
	Attenuated androgens	Oral; short course before trigger/procedure	Increase hepatic C1-INH synthesis	Oral option	Adverse effects; not preferred	Second-line STP when IV C1-INH is unavailable
Long-term prophylaxis	Plasma-derived SC C1-INH	SC; regular prophylactic dosing	Maintains functional C1-INH	Effective established prophylaxis	Ongoing injections; plasma-derived	First-line LTP
	Lanadelumab	SC; 300 mg every 2 weeks; may extend to every 4 weeks	Monoclonal antibody inhibiting plasma kallikrein	High efficacy; convenient SC dosing	Injection-site reactions; cost/access	First-line LTP
	Berotralstat	Oral; 150 mg daily with food	Oral plasma kallikrein inhibitor	Oral once-daily option	GI effects; drug interactions; dose adjustments may apply	First-line LTP
	Garadacimab	SC; 400 mg loading, then 200 mg monthly	Monoclonal antibody inhibiting FXIIa	Monthly dosing; upstream pathway target	Newer therapy; access may vary	First-line LTP
	Donidalorsen	SC; 80 mg monthly; may extend to every 8 weeks	Antisense oligonucleotide reducing prekallikrein synthesis	Targeted reduction in kallikrein pathway activity	Newer therapy; long-term data evolving	First-line LTP
	Tranexamic acid	Oral; 1000–3000 mg/day divided	Antifibrinolytic; reduces plasmin-mediated activation	Oral; may be considered in select groups	Lower efficacy; limited role in current practice	Second-line LTP when first-line therapies are unavailable, not feasible, or not tolerated
	Attenuated androgens	Oral; dose individualized	Stimulate hepatic C1-INH synthesis	Historically effective in some patients	Frequent androgenic/metabolic adverse effects	Second-line LTP when first-line therapies are unavailable, not feasible, or not tolerated

Note: Doses and approved age ranges may vary according to product labeling and regulatory region. Abbreviations: C1-INH, C1 inhibitor; FFP, fresh frozen plasma; FXIIa, activated factor XII; IV, intravenous; LTP, long-term prophylaxis; ODT, on-demand treatment; SC, subcutaneous; STP, short-term prophylaxis.

## Data Availability

No new data were generated or analyzed in this study. Data sharing is not applicable to this article as it is based on previously published literature.
